# The treatment of primary mediastinal large B-cell lymphoma: a two decades monocentric experience with 98 patients

**DOI:** 10.1186/s12885-017-3269-6

**Published:** 2017-04-17

**Authors:** Alessandro Broccoli, Beatrice Casadei, Vittorio Stefoni, Cinzia Pellegrini, Federica Quirini, Lorenzo Tonialini, Alice Morigi, Miriam Marangon, Lisa Argnani, Pier Luigi Zinzani

**Affiliations:** Institute of Haematology “L. e A. Seràgnoli”, University of Bologna, Via Massarenti, 9 – 40138 Bologna, Italy

**Keywords:** Chemotherapy, MACOP-B, primary mediastinal lymphoma, radiotherapy, rituximab

## Abstract

**Background:**

The purpose of this study is to investigate the most suitable first-line approach and the best combination treatment for primary mediastinal large B-cell lymphoma (PMLBCL) as they have been matter of debate for at least two decades.

**Methods:**

Our single centre experience in the treatment of 98 de novo PMLBCL patients over the last 20 years is reviewed. All patients received MACOP-B chemotherapy. Thirty-seven received both rituximab and mediastinal radiotherapy; 30 were irradiated after chemotherapy, although not receiving rituximab and 20 received rituximab without radiotherapy consolidation. Eleven patients received chemotherapy only.

**Results:**

Sixty-one (62.2%) patients achieved a complete response after MACOP-B (with or without rituximab); among the 27 (27.6%) partial responders, 21 obtained a complete response after radiotherapy. At the end of their scheduled treatment, 82 patients (83.7%) had a complete and 6 a partial response (6.1%). Eleven patients relapsed within the first 2 years of follow-up. The 17-year overall survival is 72.0% (15 patients died); progression-free and disease-free survival are 67.6% and 88.4%, respectively. A statistically significant difference in overall and progression-free survival was noted among treatment groups, although no disease-free survival difference was documented.

**Conclusions:**

Our data indicate that a third-generation regimen like MACOP-B could be considered a suitable first-line treatment. Mediastinal consolidation radiotherapy impacts on survival and complete response rates and remains a good strategy to convert partial into complete responses. Data suggest that radiotherapy may be avoided in patients obtaining a complete response after (immuno)chemotherapy, but this requires confirmation with further ad hoc studies.

## Background

The 1994 Revised European American Lymphoma (REAL) Classification firstly recognizes primary mediastinal large B-cell lymphoma (PMLBCL) as a subtype of diffuse large B-cell lymphoma (DLBCL), although it has been regarded as a specific clinical and biological entity since the 2001 World Health Organization classification [1, 2].

It is a rapidly-growing and progressive neoplasm, normally presenting with bulky masses usually exerting compressive effects on mediastinal structures, giving rise to the possible abrupt onset of dyspnea, dysphagia, thoracic pain, facial, neck, breasts and arms edema and pleuro-pericardial effusions. In this sense, it should be regarded as a hematological emergency, and promptly treated: initial therapy is therefore crucial for the management of this disease.

In the last 15 years, several issues have emerged regarding the treatment of this disease, and in particular: 1) the choice of initial chemotherapy approach, based on either CHOP (cyclophosphamide, doxorubicin, vincristine and prednisone) and CHOP-like schedules or more intense ones, such as the so-called “third-generation” regimens, perhaps including a high-dose consolidation with autotransplantation for patients in first remission [3]; 2) the value of a rituximab-based immunotherapy in this subset of patients, on the basis of the results obtained in randomized studies involving DLBCL patients [4, 5]; 3) the role of external beam radiotherapy (RT), as an adjuvant strategy through which consolidate a response to chemotherapy and produce an eradication of the disease [6].

In terms of first-line chemotherapy, if on the one hand the CHOP regimen has been mainly adopted by American centers, the European experience has carried out the evidence that MACOP-B (methotrexate, doxorubicin, cyclophosphamide, vincristine, bleomycin, prednisone) or VACOP-B (same as MACOP-B, with etoposide instead of methotrexate), both weekly-based “third-generation” dose-dense regimens, may be superior to CHOP [7–9]. As a consequence of the application of dose-dense regimens, in fact, remission rates and survival functions have appeared to be at least as good as – or probably even better than – those observed for DLBCL patients, thus retracting the initial impression that PMLBCL was per se a prognostically unfavorable subset of DLBCL. Although this conclusion is drawn from existing reports, no randomized clinical trial have been carried on so far. It is clear, however, that an anthracycline-containing regimen should be regarded as the first approach to PMBCL [10].

The addition of rituximab to first line chemotherapy has shown no advantages in terms of overall and relapse-free survival if the monoclonal antibody was added to a third-generation regimen [11], although R-CHOP chemoimmunotherapy seems significantly superior to CHOP alone [12, 13].

External RT, conceived as the delivery of radiation on residues of bulky masses at the end of chemotherapy, has shown a great efficacy when it was incorporated after the completion of an induction strategy based on chemotherapy, particularly in converting partial responses into complete responses and in rendering active residual masses negative at gallium scan or positron emission tomography (PET) [14–16].

This report presents our 20 years monocentric experience in the first-line treatment of primary mediastinal patients, in accordance with our Institute treatment policy and with the practical guidelines outlined by the Italian Society of Hematology [10].

## Methods

To perform this population-based retrospective study, our clinical database was searched to find all the consecutive patients with a diagnosis of PMLBCL, homogeneously treated with a third-generation MACOP-B chemotherapy regimen, regardless they received either chemotherapy alone, immunotherapy, consolidative RT, or a combination of all these strategies. Patients treated with chemotherapy regimens other than MACOP-B were excluded. The study was approved by our institutional board and by our Ethical Committee and has been performed in accordance with the ethical standards as laid down in the 1964 Declaration of Helsinki and its later amendments. Patients were consecutively enrolled to avoid selection bias, and all patients provided written informed consent to collect retrospectively their data. We obtained a special permission (for scientific purpose) from our Ethical Committee to collect even data of patients who were deceased or lost to follow up.

### Diagnostic and staging procedures

Between October 1989 and April 2010, 98 patients with de novo PMLBCL were diagnosed and subsequently treated in our Institution. Diagnostic material was obtained by supraclavicular or transthoracic lymph node biopsy, thoracotomy or mediastinoscopy. Initial clinical evaluation included physical examination, hematologic and biochemical survey, chest X-ray, computed tomography (CT) scan of neck, chest, abdomen and pelvis, and unilateral bone marrow biopsy. PET scan was also performed at baseline in all patients treated after 2001.

Disease stage was established according to the Ann Arbor staging system. Stage II indicated disease spread within contiguous thoracic, jugular or supraclavicular nodes, whereas the presence of distant, non-contiguous, involved nodes on both sides of the diaphragm was consistent with stage III. Patients with any extranodal involvement apart from mediastinal disease were categorized as stage IV. The extent of mediastinal disease was defined as mediastinal mass ratio (MMR), which was calculated by measuring the maximum single horizontal width of the mass on a standing chest radiograph, and dividing it by the maximum intrathoracic diameter. An MMR exceeding one third or a mass measuring more than 10 cm in its largest diameter as measured by CT scan was considered bulky.

### Treatment protocol

All patients were treated with the MACOP-B regimen, given for 12 consecutive weeks, with leucovorin rescue after any methotrexate-containing cycle. The median number of cycles delivered was 12. Rituximab was administered every 21 days (375 mg/m^2^) along with chemotherapy in 57 patients (58.2%), all treated after 2001, when it became available in Italy [7].

### Disease restaging, response assessment and survival analysis

Radiologic restaging was performed by total body CT scan 1 month after the end of immuno/chemotherapy, and then 3 months after the completion of RT. PET scan was done at the same timepoints, whenever available. Bone marrow biopsy was repeated only if positive at baseline. Treatment responses were categorized according to standardized response criteria [17, 18]. Nodal residues larger than 1.5 cm which have regressed by more than 75% in their major diameter were compatible with a complete response (CR), and regarded as residual scar tissue. PET negativity was corroborative of a CR.

Overall survival (OS) was calculated from diagnosis to the last follow-up or death for any cause; progression-free survival (PFS) was calculated from diagnosis to the first disease progression or death; disease-free survival (DFS) was determined in all CR patients as the time between the first documented responses and the first disease relapse, or death as a result of lymphoma or acute treatment toxicity. Survival analysis was conducted according to Kaplan-Meier’s method and log rank test was used for comparisons [19]. Demographics and patients’ characteristics were summarized by descriptive statistics and compared using χ^2^ test. Statistical analyses were performed with Stata11 (StataCorp LP, TX) and *p*-values were set at 0.05.

## Results

### Patients’ characteristics and disposition

The median age at presentation was 34.5 (range 15.7–69.5) years; 58 patients were females and 40 males, with a male-to-female ratio of 1:1.45. Three patients (3.1%) presented with stage I disease, 68 (69.4%) with stage II, 9 (9.2%) with stage III and 18 (18.4%) with stage IV disease, with lung, spleen and kidney involvement. B-symptoms were present in 41 (41.8%) patients; bulky disease was detected in 95 (96.9%) patients, with a superior vena cava syndrome in 43 (43.9%) patients.

Sixty-seven (68.4%) patients received mediastinal RT, 4 to 6 weeks after the completion of immuno/chemotherapy, with tumor doses ranging from 30 to 36 Gy over a 4 to 5 weeks treatment schedule, with fractions of 180 cGy/day for 5 days per week. The decision to use RT was based on era-specific institutional guidelines: it was routinely administered after chemotherapy in all patients since 1993 to 2002; before 1993, it was delivered upon physician’s discretion; after 2002, along with the use of PET in detecting potential residual masses after chemotherapy, RT was spared in those patients with a negative PET-scan and without bulky disease at onset.

Among the 57 patients who received rituximab, 37 (64.9%) underwent RT, whereas among the 41 who did not receive rituximab, RT was delivered in 30 (73.2%) patients. Eleven (11.2%) patients received chemotherapy only, and 37 (37.8%) received both immunotherapy and RT. According to the treatment received, patients were subdivided in 4 subgroups, as outlined in Table 1. Patients’ clinical characteristics are reported in Table 2.

**Table 1:** Patients’ subgroups according to the treatment received

Subgroup	MACOP-B	Rituximab	Radiotherapy	*n*(%)
1	Yes	No	No	11 (11.2%)
2	Yes	Yes	No	20 (20.4%)
3	Yes	Yes	Yes	37 (37.8%)
4	Yes	No	Yes	30 (30.6%)
*Total* (%)	98 (100%)	57 (58.2%)	67 (68.4%)	98 (100%)

**Table 2:** Patients’ characteristics and treatment outcomes according to subgroups

	Subgroup 1	Subgroup 2	Subgroup 3	Subgroup 4
Treatment type Patients, *n*	CHT 11	Rituximab + CHT 20	Rituximab + CHT + RT 37	CHT + RT 30
Male: female Stage I-II Stage III-IV B-symptoms LDH elevation SVC syndrome	4: 7 5 (45.5%) 6 (54.5%) 7 (63.6%) 5 (45.5%) 7 (63.6%)	7: 13 9 (45.0%) 11 (55.0%) 10 (50.0%) 5 (25.0%) 7 (35.0%)	19: 18 34 (91.9%) 3 (8.1%) 10 (27.0%) 3 (8.1%) 13 (35.1%)	10: 20 23 (76.7%) 7 (23.3%) 14 (46.7%) 12 (40.0%) 16 (53.3%)
ORR CR PR SD PD	90.9% 6 (54.5%) 4 (36.4%) 1 (9.1%) 0	55.0% 9 (45.0%) 2 (10.0%) 0 9 (45.0%)	100% 37 (100%) 0 0 0	100% 30 (100%) 0 0 0
Relapse/Progress. Deaths	2 (18.2%) 2 (18.2%)	3 (15.0%) 7 (35.0%)	2 (5.4%) 2 (5.4%)	4 (13.3%) 4 (13.3%)
9 years OS (^a^) 9 years PFS (^a^) 5 years DFS	80.0% 63.6% 66.7%	62.3% 45.0% 90.0%	93.6% 94.4% 94.4%	92.2% 86.5% 86.5%

(^a^) OS and PFS are determined at 6 years for patients in subgroup 2

### Overall treatment response and survival

**Fig. 1:**
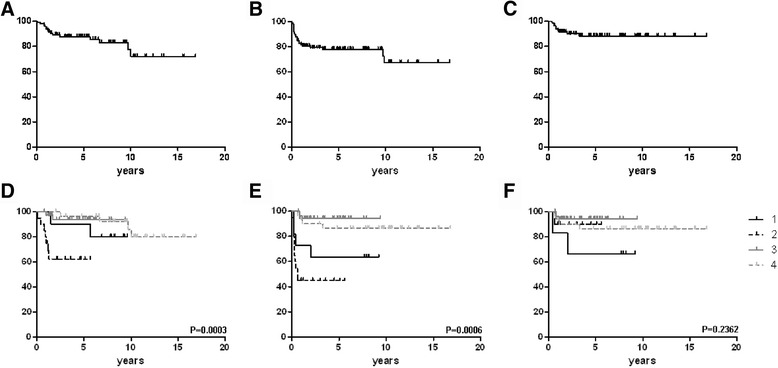
Overall survival (**a**), progression-free survival (**b**) and disease-free survival (**c**) curves plotted for the entire population on study. Subgroup survival analysis is shown underneath: overall survival (**d**), progression-free survival (**e**) and disease-free survival (**f**). *Solid black line* is for subgroup 1, *dashed black line* is for subgroup 2, *solid grey line* is for subgroup 3 and *dashed black line* is for subgroup 4. Vertical axis shows survival percentages

After 12 cycles of MACOP-B regimen (with or without rituximab), 61 patients out of 98 (62.2%) achieved a CR and 27 (27.6%) a partial response (PR); a stable disease (SD) was documented in one patient, and 9 showed progression (PD). Among those who were irradiated after immuno/chemotherapy, 21 patients previously in PR could convert their disease status to a CR, with no patients being with residual disease after RT. Upon completion of the scheduled treatment, 82 patients achieved a CR (83.7%) and 6 obtained a PR (6.1%), yielding an overall response rate (ORR) of 89.8%. At the time of writing, 73 (88.4%) patients who achieved a CR are still in continuous CR. Median follow-up duration for the entire cohort of patients is 7.6 years. The projected OS at 17 years for all the patients is 72%, with a PFS of 67.6% and a DFS of 88.4%, with all curves showing a *plateau* (Fig. 1a-c).

### Analysis of response failures

Eleven patients (11.2%) showed a disease relapse or progression during follow-up, in any case within the first 2 years after treatment completion. Nine patients were in CR after therapy and 2 in PR. Five received a rituximab-based treatment and 6 were irradiated. Salvage therapy for these patients consisted of autologous stem cell transplantation in all but one cases, with rapid disease progression and death in 6 of them. The patient who did not receive any further treatment rapidly died of disease.

Fifteen patients died during follow-up (15.3%), 13 as a consequence of lymphoma persistence after first-line treatment or disease relapse or progression. Two patients died of a secondary neoplasm (colon carcinoma) both in a CR status and more than 10 years after the conclusion of the treatment. Although both patients belonged to subgroup 4, none of the two solid tumors developed inside a previously irradiated field.

### Subgroup analysis

Clinical characteristics across the 4 subgroups were comparable, with no statistically significant differences seen at the χ^2^ test. Overall survival and PFS curves plotted for the four subgroups show a statistically significant difference (*p* = 0.0003 and *p* = 0.0006, respectively), although no difference among groups can be seen in terms of DFS (0.2362) (Fig. 1d-f). However, if OS curves for subgroup 1 and 2 are taken together – i.e. considering patients treated with chemotherapy or chemo + immunotherapy without any RT consolidation – no statistically significant difference can be detected (*p* = 0.0806), thus the discrepancy between these data does not depend either on patients’ selection or on particularly unfavorable clinical characteristics of patients in subgroup 2.

All the patients receiving RT – as a consolidation strategy after they had obtained either a CR or a PR – showed an ORR of 100%, with no residual disease being detectable after radiation. This favorable result can be appreciated both in patients receiving rituximab (subgroup 3) and in those with no exposure to the antibody (subgroup 4), with comparable OS rates in the two subgroups at 9 years (*p* = 0.5103).

DFS rates between subgroup 2 and 3 do not significantly differ (*p* = 0.55), although the number of patients in CR after treatment is substantially different. This indicates that patients in CR after chemo + immunotherapy behave similarly to those who achieve a CR after receiving mediastinal RT, suggesting that RT has a small consolidative potential in those who obtain a CR status after chemo-immunotherapy only.

### Role of rituximab

**Fig. 2:**
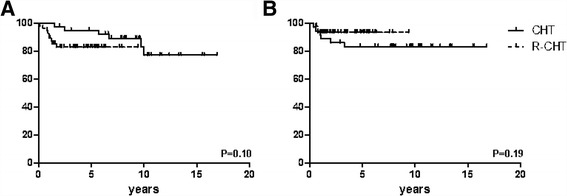
Overall survival (**a**) and disease-free survival (**b**) curves plotted according to the administration of rituximab with chemotherapy. Vertical axis shows survival percentages. *R* rituximab; *CHT* chemotherapy

When patients are subdivided according to whether they have received rituximab or not (Table 3), regardless a subsequent consolidative RT, no statistically significant differences in terms of OS and DFS (*p* = 0.1 and 0.19, respectively) can be observed (Fig. 2), thus indicating that the addition of the anti-CD20 monoclonal seems to have a limited impact on patients’ survival in our study population. Of note, however, a more favorable trend to better PFS and DFS is observed in those belonging to subgroup 3 compared to patients in subgroup 4, the former also receiving rituximab together with chemotherapy and RT consolidation.

**Table 3:** Patients’ characteristics and treatment outcomes according to exposure to rituximab

	CHT ± RT	R + CHT ± RT
Patients, *n*	41	57
Male: female Stage I-II Stage III-IV B-symptoms LDH elevation SVC syndrome	14: 27 28 (68.3%) 13 (31.7%) 21 (51.2%) 17 (41.5%) 23 (56.1%)	26: 31 43 (75.4%) 14 (24.6%) 20 (35.1%) 8 (14.0%) 20 (35.1%)
ORR CR PR SD PD	97.6% 36 (87.8%) 4 (9.8%) 1 (2.4%) 0	84.2% 46 (80.7%) 2 (3.5%) 0 9 (15.8%)
9 years OS 9 years DFS	89.0% 83.2%	83.1% 93.5%

## Discussion

The clinical and pathological peculiarities of PMLBCL, the high chances of cure documented in literature in more than 20 years of international experience and the long disease-free life-expectancy of cured patients, have always drawn attention in finding out the most suitable first-line approach and the most convenient combination of treatment modalities, on the one hand trying to maximize the long-term clinical outcomes, while on the other reducing the potential harmful consequences of highly toxic combined treatments [20, 21].

Our monocentric experience over a period of more than 20 years ideally encompasses all the issues met by treating physicians throughout the years, and may virtually suggest some still open points which require clarification with further ad hoc studies. It consists of a series of 98 patients homogeneously treated with a weekly “third-generation” schedule, who in part also received external mediastinal RT and/or anti-CD 20 immunotherapy, depending on our institutional era-specific guidelines.

“Third-generation” regimens, have become the standard of treatment in many European institutions, following the favorable results obtained when compared with the standard CHOP regimen. Lazzarino et al. documented MACOP-B/VACOP-B superiority on CHOP both in terms of CR rates and relapse-free survival (RFS), with 73% CR rate for the former versus 36% for the latter, and a 3-years RFS of 58% versus 38% [22, 23]. A retrospective multicenter Italian experience on 138 patients appeared later again emphasized the difference between the two regimens: patients on MACOP-B/VACOP-B achieved better results than those on CHOP, in terms of complete responses and event-free survival (EFS) rates, with statistically significant difference in low and low-intermediate International Prognostic Index risk groups (into which the majority of patients with PMLBCL generally fall), whereas lacking significance in high-intermediate and high-risk disease [6, 24].

The role of the anti-CD20 monoclonal antibody, rituximab, in a context of a chemo-immunotherapy regimen, still represents a matter of debate: its role in patients with PMLBCL is less well established than in DLBCL, and most of available data derive from retrospective experiences and do not rely on appropriately powered randomized trials. On the one hand, rituximab added to CHOP has demonstrated better EFS and OS rates than CHOP alone (80% and 89% versus 47% and 69% at 5 years, respectively), as well as higher complete response rates and a significant reduction of disease progression [12, 13, 21]. However, in a study from British Columbia, a comparison of rituximab-CHOP and CHOP alone in PMLBCL patients has failed to show any clear survival advantage of the former regimen [25]; moreover, patients treated with MACOP-B/VACOP-B within the same institution showed superior outcomes over CHOP-type treatments, in terms of OS (87%, 82% and 71% for patients treated with M[V]ACOP-B, rituximab-CHOP and CHOP, respectively). Data from a recent experience have also shown that in a subset of 63 PMLBCL patients treated with rituximab-CHOP, with or without radiation, a primary induction failure unacceptably occurred in 21% of the treated patients, particularly in those with increased IPI score, advanced age and stage or multiple extranodal localizations [21, 26].

A multicenter Italian experience has demonstrated that the combination of rituximab with a “third-generation” regimen is not significantly different from rituximab-CHOP, rituximab-EPOCH (etoposide, doxorubicin, cyclophosphamide, vincristine, prednisone) or M[V]ACOP-B therapy alone in terms of EFS: this means that the addition of rituximab may improve outcomes if a CHOP-like regimen is used as induction therapy, but it confers little benefit if it is added to a more intense treatment strategy [11]. Data from our series indicate no substantial improvement from the addition of rituximab to MACOP-B (subgroups 1 and 2, Table 3 and Fig. 2) in terms of OS (*p* = 0.0806 between subgroups 1 and 2; *p* = 0.10 between R-MACOP-B and MACOP-B only). The better OS and PFS outcomes seen in subgroups 3 and 4, therefore, presumably rely more on the use of RT than of rituximab.

It is clear, in fact, that external mediastinal RT, delivered after immuno/chemotherapy as a consolidative strategy, still impacts markedly on global survival and on CR rates, being able to convert PRs to CRs in the majority of cases [6, 14, 27]. In a retrospective multinational study on 426 untreated patients, data from patients treated with CHOP/CHOP-like, M[V]ACOP-B regimens and high-dose therapy/autotransplantation were compared: CR rates obtained upon completion of the three chemotherapy arms were similar (49%, 51% and 53%, respectively), but they significantly differed after mediastinal RT (67%, 84% and 77%, respectively) and in terms of 10-years OS (44%, 71% and 77%, respectively) and PFS (35%, 67% and 78%, respectively) [28].

Concerns exist, however, regarding the incidence of second malignancies and late side effects after chest irradiation, mainly on the cardiovascular system [29–31]: risks and benefits should be thoroughly balanced at the moment of treatment planning, although more experience is required in identifying patients in whom RT can be spared. Dunleavy et al. have recently demonstrated that the use of a dose-adjusted chemotherapy based on EPOCH and containing rituximab could obviate the need for radiotherapy in PMLBCL patients, with EFS and OS rates of 93% and 97%, respectively, and no relapsing patients over a median follow-up of more than 5 years [32]. However, a post-chemotherapy PET evaluation may represent a tool to guide the RT usage, reasonably sparing mediastinal irradiation in those who show a PET-negativity – i.e. Deauville score 1–2, and possibly 3 – after the completion of the chemo/immunotherapy induction [21, 33, 34]. Data in this sense are from Savage et al., where the PET-guided RT approach was applied in rituximab-CHOP-treated patients [35]. Similarly, we have recently published an experience from our institution involving 74 patients – belonging to an independent data base from the one we have described in this paper – all treated with a rituximab-MACOP-B induction therapy [36]. In both reported series, patients with a PET-documented CR were observed, whereas those with a positive PET-scan received consolidative RT [35, 36]. OS and time-to-progression seen in the first series were 89% and 83% at 5 years, respectively, whereas in our recent experience OS and DFS rates were 82% and 91% at 10 years, respectively, without any significant difference between irradiated and not-irradiated patients [35, 36]. A similar trend can be appreciated after comparing the DFS rates for subgroups 2 and 3 in the present study (90.0% and 94.4%, respectively, at 5 years, *p* = 0.55).

PET functional parameters measured at disease diagnosis may also represent useful tools in order to determine which patients will do worse with standard treatment schedules, therefore requiring a more intense approach since the beginning [37]. In addition, the detection of a truncating deletion of the *NFKBIE* gene, which encodes IκBε, a negative feedback regulator of NF-κB, and which is frequently observed in PMLBCL patients, may help segregate patients with a more aggressive disease and with therapy refractoriness [38]. It has been demonstrated that *NFKBIE*-deleted patients, in fact, display inferior outcomes compared to wild-type ones, but apparently benefit greatly from RT and rituximab [38].

## Conclusions

In conclusion, data we have gathered over a 20-year experience in the treatment of PMLBCL patients clearly indicate that: 1) a “third-generation” chemotherapy regimen such as MACOP-B is feasible and easily deliverable on an outpatient basis; 2) no statistically significant difference is seen in our series between those who received rituximab and those who did not, although a trend to better DFS rates is appreciated in patients treated with chemo + immunotherapy and RT; 3) radiotherapy in this context remains a powerful strategy to convert PRs to CRs, but it may be spared in patients obtaining a PET-documented CR after chemo-immunotherapy without any harmful prognostic consequences. Hopefully, future prospective trials – such as the ongoing International Extranodal Lymphoma Study Group 37 study – will further investigate the role of consolidation radiation therapy in PET-negative patients after induction treatment.

## Abbreviations

CHOP: Cyclophospamide, doxorubicin, vincristine and prednisone
CHT: Chemotherapy
CR: Complete response
CT: Computed tomography
DFS: Disease free survival
DLBCL: Diffuse large B-cell lymphoma
EFS: Event-free survival
EPOCH: Etoposide, doxorubicin, cyclophosphamide, vincristine, prednisone
LDH: Lactate dehydrogenase
MACOP-B: Methotrexate, doxorubicin, cyclophosphamide, vincristine, bleomycin, prednisone
MMR: Mediastinal mass ratio
NF-κB: nuclear factor κB
ORR: Overall response rate
OS: Overall survival
PD: Progressive disease
PET: Positron emission tomography
PFS: Progression free survival
PMLBCL: Primary mediastinal large B-cell lymphoma
PR: Partial response
REAL: Revised European American Lymphoma
RFS: Relapse-free survival
RT: Radiotherapy
SD: Stable disease
SVC: Superior vena cava
VACOP-B: Etoposide, doxorubicin, cyclophosphamide, vincristine, bleomycin, prednisone
